# Performance comparisons between clustering models for reconstructing NGS results from technical replicates

**DOI:** 10.3389/fgene.2023.1148147

**Published:** 2023-03-16

**Authors:** Yue Zhai, Claire Bardel, Maxime Vallée, Jean Iwaz, Pascal Roy

**Affiliations:** ^1^ Université Lyon 1, Lyon, France; ^2^ Université de Lyon, Lyon, France; ^3^ Laboratoire de Biométrie et Biologie Évolutive, Villeurbanne, France; ^4^ Service de Biostatistique-Bioinformatique, Hospices Civils de Lyon, Lyon, France; ^5^ Service de Génétique, Hospices Civils de Lyon, Bron, France; ^6^ Cellule Bioinformatique de La Plateforme de Séquençage Haut Débit NGS-HCL, Hospices Civils de Lyon, Bron, France

**Keywords:** next generating sequencing, performance evaluation, clustering model, replicate analysis, sensitivity

## Abstract

To improve the performance of individual DNA sequencing results, researchers often use replicates from the same individual and various statistical clustering models to reconstruct a high-performance callset. Here, three technical replicates of genome NA12878 were considered and five model types were compared (consensus, latent class, Gaussian mixture, Kamila–adapted k-means, and random forest) regarding four performance indicators: sensitivity, precision, accuracy, and F1-score. In comparison with no use of a combination model, i) the consensus model improved precision by 0.1%; ii) the latent class model brought 1% precision improvement (97%–98%) without compromising sensitivity (= 98.9%); iii) the Gaussian mixture model and random forest provided callsets with higher precisions (both >99%) but lower sensitivities; iv) Kamila increased precision (>99%) and kept a high sensitivity (98.8%); it showed the best overall performance. According to precision and F1-score indicators, the compared non-supervised clustering models that combine multiple callsets are able to improve sequencing performance vs. previously used supervised models. Among the models compared, the Gaussian mixture model and Kamila offered non-negligible precision and F1-score improvements. These models may be thus recommended for callset reconstruction (from either biological or technical replicates) for diagnostic or precision medicine purposes.

## 1 Introduction

Evaluating the performance of an individual’s DNA sequencing results is often hampered by the lack of gold standard. A number of researchers use then replicates of DNA sequencing results from the same individual or from monozygotic twins to reconstruct a set of high-quality calls (Zook et al., 2014). Sequencing results obtained from two or more distinct samples from a same individual are called biological replicates, whereas sequencing results obtained from two or more distinct vials of a single sample are called technical replicates (Robasky et al., 2014). Technical replicates may stem from using different sequencing platforms, different bioinformatics analysis tools, or repeated sequencing with the same platform and same bioinformatics tool. With both types of sequencing replicates, several methods have been widely used to obtain more reliable sequencing results.

Among these methods, a simple one is the concordance-based model where a “consensus” can be defined according to various degrees of agreement between callsets (Trubetskoy et al., 2015). Although this model may seem “naïve”, several investigations have suggested that its performance may not be worse than that of a machine-learning method (Wang et al., 2020).

Another method is latent class analysis (LCA) that is commonly used in biology and medicine to evaluate test performance without gold standard. In a classical latent class model, the latent variable and the observed variables are all categorical and there is a conditional independence between the observed variables within each latent class. Extensions of this classical model have been developed to account for local dependence, such as using random effects or correlation coefficients. Other extensions included covariables with effects on the latent variable or on the observed variables (Huang and Bandeen-Roche, 2004). Furthermore, Bayesian latent class analyses have been also used to provide combinations of callsets with improved performance indicators (Cantarel et al., 2014). A similar approach was the Gaussian mixture model in which the categorical latent variable is the class membership of the observations and where the observed continuous variables within each latent class follow hypothetically a Gaussian distribution. Finally, machine-learning methods (k-nearest neighbors, random forest, naïve Bayes classifier, or support vector machine) were also used to merge several callsets (Gézsi et al., 2015; Wang et al., 2020). Table 1 provides a short overview of the most relevant methods and studies designed to combine multiple callsets.

**TABLE 1:** Overview of the most relevant methods and studies designed to combine multiple callsets.

Authors	Algorithm	Model type	References
Trubetskoy et al., 2015	CGES	Consensus	Bioinformatics 2015; 31(2):187
Wang et al., 2020	SomaticCombiner	Consensus	Sci Rep 2020; 10:12898
Chiara et al., 2018	CoVaCS	Consensus	BMC Genomics 2018; 19:120
Hwang et al., 2014	---	Consensus and logistic regression	Hum Mutat 2014; 35(8):936
Cantarel et al., 2014	BAYSIC	Bayesian latent class model	BMC Bioinformatics 2014; 15:104
DePristo et al., 2011	VQSR	Gaussian mixture model	Nat Genet 2011; 43(5):491
Hwang et al., 2019	---	Gaussian-multinomial mixture model	Sci Rep 2019; 9(1):3219
Huang et al., 2019	SMuRF	Random forest	Bioinformatics 2019; 35 (17): 3157

The literature on processing replicate sequencing results is rather scanty and a number of methods do not satisfy specific research needs. This work intended to explore the main ways of dealing with multiple NGS results stemming from biological or technical replicates, investigate their properties, and compare their key performance indicators to help choosing the most performing among readily implementable methods able to improve sequencing performance. It explored the consensus model, the latent class model, the mixture model, and random forest regarding their abilities to produce a callset with improved quality. It compared their main performance indicators: precision, recall, and F1-score.

## 2 Methods

### 2.1 The study data

The present study used calling results from sequencing three technical replicates of genome NA12878. NA12878 is a human DNA sample that is “thought to represent the best-characterized diploid human genome in the world”, is “considered as a ‘reference material’ by the National Institute of Standards and Technology (NIST)”, and includes “near-perfect genome sequences for public use” as well as “truth sequences” established after repeated sequencings “using a wide variety of technologies and computational pipelines”. Today, more than 80% the NA12878 cell line’s genome is considered known with high confidence. This is why it is used as benchmark for assessing the performance of sequencing platforms or bioinformatic pipelines (Krol, 2015).

All three sequencing procedures were carried out on Illumina NovaSeq 6000 system platform. The samples were then aligned with Burrow-Wheeler Aligner (BWA-MEM) (Li, 2013) against the GRCh37 version of the human reference genome. Genome Analysis Toolkit (GATK) duplicate marking, base quality score recalibration, and indel realignment were applied (McKenna et al., 2010). The resulting sequencing data were deposited in the European Nucleotide Archive.

Variant calling was performed by joint genotyping according to the GATK Best Practices recommendations (DePristo et al., 2011; van der Auwera and O’Connor, 2020). Concordance rates between the calling results of the replicates were calculated. The concordance rate was defined as the number of sites called in the same category (see 2.2) by each replicate divided by the total number of sites called as variants by at least one of the replicates.

The latest version (v 4.2.1) of Genome in a Bottle (GIAB) variant calling benchmark set was used as ‘gold standard’ (Zook et al., 2016; Wagner et al., 2022). This version has a higher coverage of the GRCh37 reference genome and includes more difficult-to-map regions than the previous version (Wagner et al., 2022).

### 2.2 Basic definitions and main covariables

Performance considered only bps from the GIAB benchmark region, each bp position being a statistical unit and each GIAB benchmark result a true status of each bp. Here, only performance in single nucleotide variant (SNV) analysis was considered.

In this analysis, the variant calling results in the VCF file and the GIAB benchmark callset (gold standard set) were considered to belong to one of three categories: homozygous reference, heterozygous variants, and homozygous variants. A true positive (TP) was defined as a variant call in the query callset that belongs to the same category as in the gold standard set; i.e., both are heterozygous variants or both homozygous variants despite potential allele or phasing differences. A false negative (FN) was defined as a variant in the gold standard set called as non-variant in the query callset. A false positive (FP) was defined as a non-variant in the gold standard set called as variant in the query callset or a variant in the gold standard set called as variant in a different category. A true negative (TN) was defined as a non-variant in the gold standard set called as non-variant in the query callset. No-calls in the VCF file were considered as non-variants. This recalls the “genotype match, for which only sites with matching alleles and genotypes are counted as TPs” (Krusche et al., 2019), though, in this study, the criteria for true positivity were less stringent.

The covariables included in the models were:

1) The depth of coverage (DP); i.e., the number of informatics reads covering a given base-pair. In this study, the mean DP value across the three replicates was circa 38 and the DP value ranged from 0 to 13,858.
2) The allele balance (AB; i.e., the number of reads supporting the alternative allele divided by the number of all informatics reads at a specific site) ranged from 0 to 1.
3) The QualByDepth (QD); i.e., the site-level Phred-scaled confidence for the existence of variant divided by the number of reads supporting the alternative allele in variant samples. Here, the QD value ranged from 0.02 to 42.9.
4) The genotype quality (GQ); i.e., the Phred-scaled confidence for the called genotype (ranged from 0 to 99).
5) The mapping quality (MQ); i.e., the root mean square of the MQ of reads across all samples (ranged from 20 to 60).

Covariates DP, AB, and GQ were obtained from the VCF file for each bp in each sample (here, replicate), and then the mean of each of the three values was calculated. MQ and QD were obtained from the VCF file for each bp and had the same values across the three samples.

### 2.3 Clustering models used for NGS reconstruction

Five types of models were selected for reconstructing NGS result from technical replicates.

#### 2.3.1 The consensus (or concordance-based) model

In this model, “strict consensus” was considered whenever all variant calling results across all replicates agreed and “majority consensus” whenever there was a majority of variant calling results across all replicates (Trubetskoy et al., 2015; Wang et al., 2020). Here, it is the majority consensus that was used. In case of no majority consensus, the sites were classified as homozygous variants.

#### 2.3.2 The latent class model without covariables

This type of analysis was often used to evaluate the performance of diagnostic tests in the absence of gold standard. A latent class analysis is a mixture model where both the observed and unobserved variables are categorical. A classical LCA assumes conditional independence between observed variables (here, called genotype categories) given the latent class (here, the true genotype status).

Let *i* represent each site in the VCF file, *r* the latent classes 1 to 3. $Y_{i}$ represents the calling results in replicates 1 to 3 for site *i* ( $Y_{1}$ , $Y_{2}$ , and $Y_{3}$ are categorical variables with three categories that correspond to the three genotype categories). $p_{r}$ denotes the prevalence of latent class *r*. $\pi_{r}\left(Y_{1}\right),\pi_{r}\left(Y_{2}\right)$ , and $\pi_{r}\left(Y_{3}\right)$ are the probability mass functions of variables $Y_{1}$ , $Y_{2}$ , and $Y_{3}$ for latent class *r*.

The equation of this model may be written:

$$P\left(Y_{i}|p,\pi \right)=\overset{3}{\underset{r=1}{\sum }}p_{r}\times \pi_{r}\left(Y_{1}\right)\times \pi_{r}\left(Y_{2}\right)\times \pi_{r}\left(Y_{3}\right)$$ (1)

The model parameters, namely, $p_{r}$ and $\pi_{jrk}$ , were estimated with an expectation-maximization (EM) algorithm using 50 sets of random initial values.

#### 2.3.3 The latent class model with covariables

In this model, covariables’ effects were put on the prior probability of class membership ( $P_{r}$ in Eq. 1) and modelled using a logistic link. Covariables that are potentially correlated with the latent bp status were included; namely, Allele Balance (AB; the mean AB value of the three replicates), QualByDepth (QD), and Mapping Quality (MAPQ). Univariate models were first fitted for each covariable, then models were fitted with all possible pairs of covariables. Model parameters $\left(\pi ,p\right)$ were estimated using 100 sets of random initial values. Models with distinct covariables were compared with the Bayesian information criterion (BIC) as a measure of model fit.

The latent class model without covariables and the latent class model with covariables were fitted using package “poLCA” (v. 1.6.0.1) in R (v. 4.1.3) (Linzer and Lewis, 2011).

#### 2.3.4 The Gaussian mixture model

The Gaussian mixture model assumes that the observed variables within each latent class follow a multivariate normal distribution. Here, it is the observed continuous covariables that were modelled; the calling results of each replicate were not included. The covariables included in the model were read depth (DP; the mean DP value of the three replicates), allele balance (AB; the mean AB value of the three replicates), and quality by depth (QD); all were assumed to be normally distributed.

Let $x_{i}$ denote the vector of covariables for site *i*, $p_{r}$ the prevalence of each latent class (*r =* 1, 2, or 3), $\alpha_{r}$ the parameters of the multivariate normal distribution for latent class *r*. $h\left(x_{i}|\alpha_{r}\right)$ is the probability density function for latent class *r*, with parameters $\alpha_{r}$ . Thus, the probability density function for $x_{i}$ can be written:

$$f\left(x_{i}|p,\alpha \right)=\overset{R}{\underset{r=1}{\sum }}p_{r}h\left(x_{i}|\alpha_{r}\right)$$

The model parameters, namely, p and α , were estimated with an expectation-maximization (EM) algorithm. This model was fitted using package “mclust” (v. 6.0.0) in R (v. 4.1.3) (Scrucca et al., 2016).

#### 2.3.5 Kamila model (k-means for mixed large datasets)

Kamila is a model-based adaptation of the k-means clustering algorithm for heterogeneous variables (mix of categorical and continuous). It uses a kernel density estimation technique to model flexibly spherical clusters in the continuous domain and uses a multinomial model in the categorical domain (Foss et al., 2016). The model parameters were estimated with an iterative process similar to an EM algorithm. One advantage of this model is to include both types of variables at the same time without pre-specifying the weights of continuous *versus* categorical variables.

The categorical covariables included were: the calling results of the three replicates and a binary covariable to indicate whether a site is present in a “difficult region” (Amemiya et al., 2019). The continuous covariables included were DP, AB, and QD. The algorithm is sensitive to outliers because it uses kernel density estimation and Euclidean distance for continuous covariables. Here, the maximum value of DP was set to 150.

This model was applied with package “Kamila” (v. 0.1.2) in R (v. 4.1.3) (Foss and Markatou, 2018).

#### 2.3.6 The random forest

An unsupervised version of the random forest model for clustering was implemented (Shi and Horvath, 2006). The algorithm started with an unsupervised random forest model to generate a synthetic dataset without correlation between covariables, and then classified the observations into the synthetic or the original dataset using a classical random forest. This generates a proximity matrix that represents the number of times observations were classified into the correct dataset. A hierarchical clustering was then applied using the proximity scores as dissimilarity measure between observations.

This model was applied with Package ‘RandomForest’ (v. 4.7-1.1) in R (v. 4.1.3) (Liaw and Wiener, 2002). Because this model is computationally expensive, only 10,000 sites from the VCF file were sampled for its use. The number of trees used was 1000.

### 2.4 Clustering choices

Among the six above-mentioned models, five generate clusters. As the purpose was identifying the three latent classes that correspond to the three genotype categories, the number of clusters in each model was fixed to three. The largest cluster had to correspond to the heterozygous variants, the intermediate cluster to the homozygous variants, and the smallest cluster to the homozygous reference. Also, any model that showed any cluster with <0.1% of the observations was considered unable to identify three clusters, and therefore not retained. This choice was made according to a prior knowledge about the relatively stable proportions of the three categories in a VCF file of WGS. The ratio of heterozygous variants to homozygous variants in the VCF files is expected to be around 2 (Guo et al., 2014; Wang et al., 2015). The reference sites (i.e., the false positives for at least one replicate) occupy usually 0.1%–10% in WGS data (Zhao et al., 2020).

### 2.5 Model result comparisons

Each callset was compared against the GIAB gold standard set. This comparison used the above-provided definitions of TPs, FPs, FNs, and TNs as well as the following performance indicators:

i) Accuracy (or 1−the overall classification error rate) was calculated as (TPs + TNs)/(TPs + FPs + FNs + TNs); i.e., over the total number of sites in the VCF file;
ii) Recall (or sensitivity) was calculated as TPs/(TPs + FNs);
iii) Precision (or positive predictive value, PPV) was calculated as TPs/(TPs + FPs);
iv) F1-score was calculated as 2 × recall × precision/(recall + precision).

All callsets (except the one generated from the random forest) included all sites in the VCF file. For the random forest callset, the total number of real variants was estimated as the number of variants in the gold standard set multiplied by the sampling proportion.

## 3 Results

### 3.1 Performance indicators for calling results of individual replicates

The precisions relative to the three replicates (1 to 3) had very close values (96.7%–96.9%) and the sensitivities were nearly the same (∼98.9%) (Table 2). The concordance rates of Replicate 1 vs. Replicates 2 and 3 were 98.4% and 98.3%, respectively; whereas the concordance rate of Replicate 1 vs. Replicate 3 was 98.2%. The concordance rate across the three replicates was 97.5%.

**TABLE 2:** Performance indicators of the clustering models under study.

Clustering model	Accuracy	Precision	Recall	F1-score
None	96.7%–96.9%	96.7%–96.9%	98.9%	97.8%–97.9%
Majority consensus	97.0%	97.0%	98.9%	97.9%
Latent class analysis without covariables	97.8%	97.9%	98.8%	98.3%
Latent class analysis with covariables	98.0%	98.0%	98.9%	98.4%
Gaussian mixture model	98.5%	99.3%	98.2%	98.7%
Kamila	99.0%	99.2%	98.8%	99.0%
Random Forest	98.2%	99.5%	97.9%	98.7%

Thus, as expected, the three replicates had similar performance indicators and there were high concordance rates between replicates. However, given the number of total loci in the VCF file (*n* = 3,351,415), the number of discordant sites across replicates was not negligible (*n* = 84,753).

Among the concordant sites across the three replicates, precision differed for different genotype categories. For the concordant heterozygous variant sites (*n* = 1,993,116), the precision was 96.8%. For the concordant homozygous variant sites (*n* = 1,273,546), the precision was 99.6%. Among the discordant sites, 55.9% were homozygous references, 39.6% heterozygous variants, and 4.5% homozygous variants in the gold standard.

### 3.2 Comparison of model fits

In this study, the five types of models used neither the same amount of information nor the same type of covariables: i) the consensus model and the classical latent class model used the categorical variant calling results from the three replicates; ii) the Gaussian mixture model used continuous covariables; iii) the latent class model with covariables, Kamila model, and random forest used categorical variant calling results as well as categorical or continuous covariables. It was therefore difficult to compare directly model fits across model types. This section presents only comparisons within each model type.

With the latent class models with one covariable (AB, QD, or MAPQ), the effect of each covariable was significantly different from 0. The model with AB showed the smallest BIC and was therefore considered as the most fitted to the data.

With the latent class model with two covariables, among the three models relative to the three pairs of covariables, the model with AB and QD had the lowest BIC. Here, it is useful to note that, with some models, the estimations of the parameters of the latent class model with covariables were not stable. With some models, the global maxima of the log-likelihood were reached in only 10% of estimation attempts. The most frequent local maxima were seldom the global maxima and the estimated proportions of heterozygous variant, homozygous variant, and homozygous reference sites were substantially different between estimation attempts. Therefore, a large number of sets of random initial values (100 rather than 50) were necessary to avoid local maxima. (Supplementary Table S1).

With the Gaussian mixture model, the chosen model (the one with the lowest BIC) was the model with three covariables: DP, AB, and QD.

### 3.3 Performance comparisons

**FIGURE 1:**
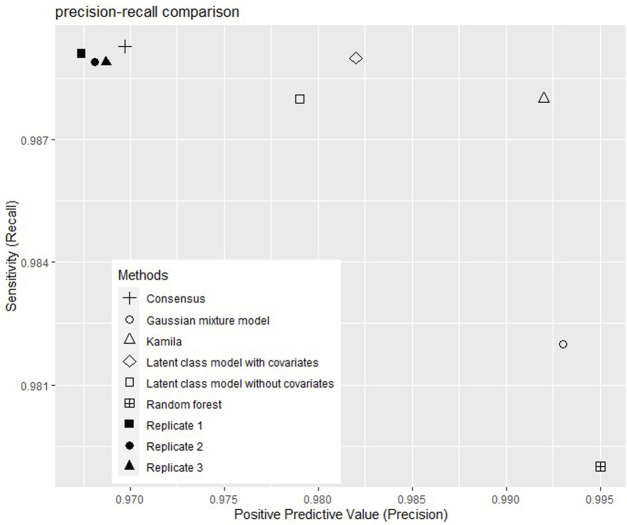
Positive predictive values and sensitivities of callsets without and with selected clustering models.

The performance indicators (accuracy, precision, recall, and F1-score) of the models are shown in Table 2 and Figure 1 shows the precision and the recalls of callsets of individual replicates and clustering models. The consensus method improved the precision by 0.1% without much decrease of the recall. Among the five clustering models, the Gaussian mixture model showed the highest accuracy (98.5%). The random forest model showed the highest precision (99.6%) but the lowest recall (98.2%). The consensus model and the latent class model with covariables showed the highest recall (98.9%). The Gaussian mixture model and random forest had high F1-scores (98.7%). Kamila model showed the highest F1-score (99.0%).

The proportions of the three genotype categories in each callset, including the gold standard GIAB benchmark set, are shown in Table 3 (Total loci: 3,351,415 in the VCF file). The first row shows the “true” category proportions in the GIAB benchmark set for all sites in the VCF file. More than 4% were classified as reference sites in GIAB set, which corresponds to the marginal false positive rate in the VCF file. Rows 2 to 5 show the proportions in the three replicates and the consensus callset. With the model-based methods (rows 6–10), these proportions were the estimated latent-class proportions. The callsets generated by the clustering models grouped more sites into the smallest class (interpreted as reference; thus, false positives) than into the consensus callset; this explains the improved precision of these models. With the Gaussian mixture model, the highest proportion was found in the reference category, which explains its higher precision and lower recall *versus* the other models.

**TABLE 3:** Proportions of the three genotype categories in each callset.

	Callset	Homozygous References (%)	Heterozygous variants (%)	Homozygous variants (%)
1	Gold standard (GIAB)	4.241	57.891	37.868
2	Calling results of Replicate 1	1.064	60.800	38.136
3	Calling results of Replicate 2	1.230	60.672	38.098
4	Calling results of Replicate 3	1.295	60.618	38.087
5	Majority consensus	1.287	60.586	38.127
6	Latent class analysis without covariables	2.283	59.596	38.121
7	Latent class analysis with covariables	2.632	59.171	38.197
8	Gaussian mixture model	4.426	58.001	37.573
9	Kamila	3.586	58.310	38.104
10	Random forest	5.560	57.440	37.000

## 4 Discussion

In this study, six clustering algorithms were run on real sequencing replicates of the NA12878 genome to compare their abilities in allowing reconstruction of a new callset with improved performance: one consensus model, two latent-class models, a Gaussian mixture model, a Kamila (adapted k-means) model, and a random forest model. These models showed various advantages. For example, the consensus model improved slightly the precision (by 0.1%) whereas the latent class model provided a non-negligible 1% precision improvement (97% to 98%) without compromising recall (98.9%). In comparison with no use of a clustering model, all six models brought ≥1% gain in sensitivity, which is not negligible: i) the Gaussian mixture and the random forest models provided callsets with high precision (>99%) but at the price of lower recall; ii) Kamila increased precision (99.2%) and kept a high recall (98.8%); it proved having the best overall performance.

In this work, the models were chosen to represent a range of major clustering models, from the most naïve (consensus) to the most sophisticated machine-learning type (random forest). One interest of this choice is that all models may be readily implemented with packages in R software. However, here, only non-supervised clustering models were compared and not supervised ones because the latter need high-quality training data (Sandmann et al., 2018) which are not usually available in clinical practice settings. The models dealt with by BAYSIC and SomaticCombiner or their equivalents were actually considered in this article as latent class model and consensus model, respectively. Indeed, in this work, the former algorithm was not considered because its results would be quite similar to those obtained with a classical latent class model and the latter is based on an approach that is close to the consensus model.

Most of the models considered here have been previously used for similar purposes; i.e., merging several either constitutional or somatic variant calling results to obtain a new callset with better performance indicators (precision or recall). Previous authors used: i) the consensus model (Hwang et al., 2014; Trubetskoy et al., 2015; Chiara et al., 2018; Di Nanni et al., 2019); ii) the Bayesian latent class model (Cantarel et al., 2014); iii) the Gaussian mixture model (DePristo et al., 2011; Hwang et al., 2019); iv) random forest (Huang et al., 2019; Wang et al., 2020). However, though usual, these models have been rarely compared, their comparison results often unclear, and the final conclusions controversial. For example, the random-forest-based ensemble caller for somatic mutation has obtained higher F1-scores than the simple consensus approach (Huang et al., 2019); however, in a study by Wang et al. (Wang et al., 2020), the authors observed that the consensus method was more robust and stable than supervised machine-learning models. They suggested that the difference between the training data and the test data contributed to the poor generalizability of machine-leaning models. In another research on the NA12878 genome that used the GIAB benchmark set as gold standard, a two-component mixture-model-based method that considered results from 70 pipelines did not significantly improve performance in terms of precision at the highest analytical sensitivity achievable vs. the highest performance of a single pipeline. However, the method led to performance improvement with another gold standard set from the ‘1000 Genomes Project’ (Hwang et al., 2019).

The models compared here did not include the same number of variables because of the hypotheses inherent to each model. Some require only continuous variables (e.g., the Gaussian mixture model), whereas others require only categorical variables (e.g., the latent class model). Thus, performance comparisons between new callsets generated by different models should be interpreted with this difference in mind. For example, Kamila and random forest models are able to include more covariables than the other models. In future works, comparisons between models with same covariables would be welcome. One current aim was to use information already available in a VCF file; however, the possibility of including more covariables may be interesting too.

In some previous research works, sites in the VCF file of presumably very low quality were filtered out before applying merging methods; i.e., a small number of sites were considered as false positives and thus excluded (Sandmann et al., 2018). Here, no sites were filtered out (all sites from the VCF file were included in the models); this allowed a more objective evaluation of the overall performance of each model. However, this choice introduced some difficulties due to the extreme values of certain variables. For example, DP has typically a long-tailed distribution and the presence of extremely high values is often an indicator of sequencing artifacts, alignment artifacts, or copy number variations (O’Rawe et al., 2013; Guo et al., 2014; Li, 2014). In common practice, the solution to extreme DP values is to exclude sites with values higher than a threshold defined according to various formulas that use the mean and standard deviation of DPs (Li et al., 2018; Pan et al., 2022); for example, a threshold 120 in the hard filters recommended by the GATK (van der Auwera and O’Connor, 2020).

In the present work, the mean DP across the three replicates was circa 38 and its maximum 13,858 and, among the compared models, Kamila is known to be relatively sensitive to extreme values because it minimizes a dissimilarity measure that is partially based on Euclidean distance in the case of continuous variables. This might explain why it failed to identify the three clusters with acceptable proportions. Indeed, the model grouped a small number of sites with extremely high DP values into one cluster (*n* = 254; i.e., 0.008% of all sites) and, as stated in 2.4, models that led to any cluster with <0.1% of the sites were considered unable to identify three clusters and thus not retained. One way to address this issue is to add one more cluster in the model (4 instead of 3). However, in this work, only three clusters were considered to allow model performance comparisons and allow each cluster to represent each genotype category. Therefore, with Kamila, the maximum DP value was set at 150 and higher values grouped together at 150. The other models that involved DP (i.e., the Gaussian mixture model and the random forest model) performed well despite the presence of high DP values (these were not then filtered out).

This study focused on the VCF file (i.e., on all sites called as variants in at least one replicate) and not on all three billion bp positions across the human genome. This is one reason for which the indicators of performance kept were only recall and precision (specificity was ignored). There are also two other practical reasons: i) negative sites are much more numerous (almost 1000 times the number of sites in the VCF file) and contain less information; thus, using them is computationally expensive and adds little information; ii) researchers, especially practitioners and lab professionals, usually use only the VCF file for routine analyses; thus, a model that requires information from the BAM file for sites called as ‘reference’ would not be practical.

One limitation of this study is that it evaluated only callsets’ performance regarding SNVs. Further studies are worth being conducted to evaluate the performance of clustering models regarding copy number variations and structural variations. Also, except for Kamila, the study included only the most classical model from each clustering algorithm type. Some model features may prove more adapted to the distribution of the variables or have more convenient underlying hypotheses. For example, latent class models that relax the conditional independence between observed variables through correlation, random effects, or covariables with effects on the class-conditional probabilities.

The Gaussian mixture model used here showed good performance vs. the other five models. However, all components of a variable distribution might not be Gaussian. For example, i) the distribution of allele balance has been already modelled using a mixture of 0-inflated beta distribution, binomial distribution, and 1-inflated beta distribution for the homozygous reference, heterozygous variant, and homozygous reference categories, respectively (Muyas et al., 2019); ii) to take into account heavytails, read depth distributions have been modelled using a compound Poisson distribution, a negative binomial distribution, or a log-normal distribution (Robinson et al., 2010; Daley and Smith, 2014; Deng et al., 2020).

From a theoretical viewpoint, a very recent article by Dang et al. (Dang et al., 2023) reviewed a selection of “mixture models that can deal with varying cluster tail-weight, skewness and/or concentration, and kurtosis” (e.g., mixtures of multivariate t-distributions, mixtures of skew-t distributions, mixtures of normal inverse Gaussian distributions, etc.). Furthermore, these authors introduced a multivariate skewed power exponential distribution that “allow for robust mixture models for clustering with skewed or symmetric components” and “model components with varying levels of peakedness, skewness, and tail-weight (light, heavy, Gaussian)”. In practice, the use of multivariate non-Gaussian mixture models is often difficult because of identifiability issues and the instability of parameter estimation. This might explain the rarity of applications on real data, which is worth being explored. We especially hope an exploration of the appropriateness of the above-mentioned models within the context of WGS data.

## 5 Conclusion

In this study, several clustering models were evaluated within the context of combining callsets from DNA sequencing replicates. These non-supervised clustering models proved able to improve sequencing performance in terms of precision and F1-score, which is comparable to what is reported about supervised models. Among the models compared here, the Gaussian mixture model and Kamila offered improvements that made precision higher than 99% and F1-score close to 99%. These models may then be recommended to reconstruct new high-performance callsets from NGS replicates. This is of particular interest for diagnosis or precision medicine whenever DNA sequencing results stem from either biological replicates (more than one sample) or technological replicates (more than one sequencing platform or analysis pipeline).

## Data Availability

The original contributions presented in the study are publicly available. This data can be found here: https://www.ebi.ac.uk/ena/browser/home. Accession number: PRJEB60499.
