# Supplementary material for: Performance comparisons between clustering models for reconstructing NGS results from technical replicates
Source: Front Genet. 2023 Mar 16;14:1148147. doi: 10.3389/fgene.2023.1148147 (PMC10060969; doi:10.3389/fgene.2023.1148147)
Supplement: Supplementary file 1 [file Table1.DOCX]

**Supplementary Material**

**Supplementary Table 1 --** Stability of the estimates obtained with the latent class analysis model with covariable QualByDepth. The model was fitted 1000 times using 1000 random initial values. The table shows the five most frequent maximum log-likelihood estimations.

| Maximum log-likelihood | Number of occurrences | Estimated latent class proportion (%) | | |
| --- | --- | --- | --- | --- |
| -779028 | 818 | 61.626 | 38.090 | 0.28382 |
| -447774* | 36 | 59.171 | 38.197 | 2.6323 |
| -794167 | 34 | 59.078 | 38.169 | 2.7520 |
| -802348 | 28 | 61.746 | 38.154 | 0.099742 |
| -805528 | 27 | 61.814 | 38.185 | 2.5314 × 10^-7^ |

* Global maximum log-likehood

**Supplementary Table 2 --** Stability of the estimates obtained with the latent class analysis model with covariable Allele Balance. The model was fitted 1000 times using 1000 random initial values. The table shows the five most frequent maximum log-likelihood estimations.

| Maximum log-likelihood | Number of occurrences | Estimated latent class proportion (%) | | |
| --- | --- | --- | --- | --- |
| -281366* | 241 | 59.365 | 38.090 | 2.5436 |
| -697800 | 205 | 61.910 | 38.090 | 3.3126 × 10^-7^ |
| -671834 | 189 | 61.868 | 37.972 | 0.15996 |
| -282909 | 160 | 59.364 | 38.059 | 2.5764 |
| -672082 | 66 | 61.831 | 37.979 | 0.18954 |

* Global maximum log-likehood
