# Supplementary material for: Performance comparisons between clustering models for reconstructing NGS results from technical replicates
Source: Front Genet. 2023 Mar 16;14:1148147. doi: 10.3389/fgene.2023.1148147 (PMC10060969; doi:10.3389/fgene.2023.1148147)
Supplement: Supplementary file 2 [file DataSheet1.docx]

## R Code ##

##

## Read VCF file ##

library(vcfR)

vcf1<- read.vcfR("..\\.snv.vcf",verbose = TRUE )

vcf1 <- addID(vcf1)

### extract vovariables and genotypes

DP <- extract.gt(vcf1, element='DP', as.numeric = TRUE)

GT <- extract.gt(vcf1, element='GT', as.numeric = FALSE)

GQ <- extract.gt(vcf1, element='GQ', as.numeric = TRUE)

AD<- extract.gt(vcf1, element='AD', as.numeric = FALSE)

MQ <-extract.info(vcf1, element = "MQ", as.numeric = TRUE)

QD<- extract.info(vcf1, element = "QD", as.numeric = TRUE)

vcf1 <- getFIX(vcf1)

vcf1 <- cbind(vcf1[, c("CHROM", "POS", "ID","REF","ALT","QUAL","FILTER")],

DP, GT, GQ, AD,MQ,QD)

colnames(vcf1) <- c("CHROM", "POS", "ID", "REF","ALT","QUAL","FILTER","DP1","DP2","DP3",

"GT1","GT2","GT3", "GQ1","GQ2","GQ3",

"AD1","AD2","AD3","MQ","QD")

#### separate SNP ###

vcf1 <- vcf1[which(vcf1[, "REF" ] == "A" |vcf1[, "REF" ] == "T"|vcf1[, "REF" ] == "C"|

vcf1[, "REF" ] == "G" ) , ]

vcf <- vcf1[which(vcf1[, "ALT" ] == "A" |vcf1[, "ALT" ] == "T"|vcf1[, "ALT" ] == "C"|

vcf1[, "ALT" ] == "G" ), ]

#### Group Genotypes into three categories ###

GT1 <- vcf1[ ,"GT1"]

GT2 <- vcf1[ ,"GT2"]

GT3 <- vcf1[ ,"GT3"]

table(GT1)

GT1[GT1 =="0/0"] <- 0

GT1[GT1 =="0|0"] <- 0

GT1[GT1 =="0/1"] <- 1

GT1[GT1 =="0|1"] <- 1

GT1[GT1 =="1/1"] <- 2

GT1[GT1 =="1|1"] <- 2

#######################

GT2[GT2 =="0/0"] <- 0

GT2[GT2 =="0|0"] <- 0

GT2[GT2 =="0/1"] <- 1

GT2[GT2 =="0|1"] <- 1

GT2[GT2 =="1/1"] <- 2

GT2[GT2 =="1|1"] <- 2

#######################

GT3[GT3 =="0/0"] <- 0

GT3[GT3 =="0|0"] <- 0

GT3[GT3 =="0/1"] <- 1

GT3[GT3 =="0|1"] <- 1

GT3[GT3 =="1/1"] <- 2

GT3[GT3 =="1|1"] <- 2

table(GT1)

### build a complete dataframe ###

vcf1 <- cbind(vcf1,GT1,GT2,GT3)

colnames(vcf1) <- c("CHROM", "POS", "ID", "REF","ALT","QUAL","FILTER","DP1","DP2","DP3",

"GT1","GT2","GT3", "GQ1","GQ2","GQ3",

"AD1","AD2","AD3","MQ","QD", "GTtri1","GTtri2","GTtri3")

## load benchmark set from GIAB ###

#########################

# giab <- read.vcfR("..\\HG001_GRCh37_GIAB_highconf_ID.vcf", verbose = TRUE )

### get covariables ###

giabGT <- extract.gt(giab, element='GT', as.numeric = FALSE)

addID(giab)

giab <- getFIX(giab)

#### separate SNP ###

giab <- giab[which(giab[, "REF" ] == "A" |giab[, "REF" ] == "T"|giab[, "REF" ] == "C"|giab[, "REF" ] == "G" ) , ]

giab <-giab[which(giab[, "ALT" ] == "A" |giab[, "ALT" ] == "T"|giab[, "ALT" ] == "C"|giab[, "ALT" ] == "G" ), ]

giabGT[giabGT =="0/1"] <- 1

giabGT[giabGT =="1/0"] <- 1

giabGT[giabGT =="1/1"] <- 2

giab <- cbind(giab[, c("CHROM", "POS", "REF","ALT")],giabGT)

### Add "GIAB" colomn in the dataframe ##

GIAB <- rep(0,nrow(vcf))

vcf<-cbind(vcf, GIAB)

vcf[which(vcf[,"ID"]%in% giab[,"ID"]), "GIAB"] <-

giab[which(vcf[,"ID"]%in% giab[,"ID"]), "GT"]

### Add mean of allele balance and DP ##

ABmean <- (vcf[,"AB1"] + vcf[,"AB2"] + vcf[,"AB3"])/3

ABmean[is.na(ABmean)] <- 0

DPmean <- (vcf[,"DP1"] + vcf[,"DP2"] + vcf[,"DP3"])/3

vcf<-cbind(vcf,ABmean, DPmean)

#########################################

### Comparison of different methods ####

#########################################

#### replace NAs ####

library(dplyr)

datalca <- datalca %>% replace(is.na(.), 0)

################### MODEL 1 ##########################

############### CONSENSUS #######################

vcf[ , "GTtri1"] <- vcf[ , "GTtri1"] +1

vcf[ , "GTtri2"] <- vcf[ , "GTtri2"] +1

vcf[ , "GTtri3"] <- vcf[ , "GTtri3"] +1

temp<- data.frame(vcf[,"GTtri1"],vcf[,"GTtri2"],vcf[,"GTtri3"], vcf[,"GIAB"])

## Different combinaisons of genotype category: ##

n010<-rbind(temp[which(temp[,1]==1 & temp[,2]==2 & temp[,3]==1), ],

temp[which(temp[,1]==1 & temp[,2]==1 & temp[,3]==2), ],

temp[which(temp[,1]==2 & temp[,2]==1 & temp[,3]==1), ])

n020<-rbind(temp[which(temp[,1]==1 & temp[,2]==3 & temp[,3]==1), ],

temp[which(temp[,1]==1 & temp[,2]==1 & temp[,3]==3), ],

temp[which(temp[,1]==3 & temp[,2]==1 & temp[,3]==1), ])

n011<-rbind(temp[which(temp[,1]==1 & temp[,2]==2 & temp[,3]==2), ],

temp[which(temp[,1]==2 & temp[,2]==1 & temp[,3]==2), ],

temp[which(temp[,1]==2 & temp[,2]==2 & temp[,3]==1), ])

n012<-rbind(temp[which(temp[,1]==1 & temp[,2]==2 & temp[,3]==3), ],temp[which(temp[,1]==1 & temp[,2]==3 & temp[,3]==2), ],

temp[which(temp[,1]==2 & temp[,2]==1 & temp[,3]==3), ],temp[which(temp[,1]==3 & temp[,2]==1 & temp[,3]==2), ],

temp[which(temp[,1]==2 & temp[,2]==3 & temp[,3]==1), ],temp[which(temp[,1]==3 & temp[,2]==2 & temp[,3]==1), ])

n022<-rbind(temp[which(temp[,1]==1 & temp[,2]==3 & temp[,3]==3), ],

temp[which(temp[,1]==3 & temp[,2]==1 & temp[,3]==3), ],

temp[which(temp[,1]==3 & temp[,2]==3 & temp[,3]==1), ])

n111<-temp[which(temp[,1]==2 & temp[,2]==2 & temp[,3]==2), ]

n112<-rbind(temp[which(temp[,1]==3 & temp[,2]==2 & temp[,3]==2), ],

temp[which(temp[,1]==2 & temp[,2]==3 & temp[,3]==2), ],

temp[which(temp[,1]==2 & temp[,2]==2 & temp[,3]==3), ])

n122<-rbind(temp[which(temp[,1]==3 & temp[,2]==3 & temp[,3]==2), ],

temp[which(temp[,1]==2 & temp[,2]==3 & temp[,3]==3), ],

temp[which(temp[,1]==3 & temp[,2]==2 & temp[,3]==3), ])

n222<-temp[which(temp[,1]==3 & temp[,2]==3 & temp[,3]==3), ]

## Attribution of consensus decision ##

temp <- cbind(temp, rep(NA, nrow(vcf)))

temp[which(rownames(temp) %in% rownames(n010)), 5] <- 0

temp[which(rownames(temp) %in% rownames(n020)), 5] <- 0

temp[which(rownames(temp) %in% rownames(n011)), 5] <- 1

temp[which(rownames(temp) %in% rownames(n012)), 5] <- 2

temp[which(rownames(temp) %in% rownames(n022)), 5] <- 2

temp[which(rownames(temp) %in% rownames(n111)), 5] <- 1

temp[which(rownames(temp) %in% rownames(n112)), 5] <- 1

temp[which(rownames(temp) %in% rownames(n122)), 5] <- 2

temp[which(rownames(temp) %in% rownames(n222)), 5] <- 2

colnames(temp)<-c("GTtri1","GTtri2","GTtri3","GIAB", "Consensus")

tabbcon <- table(temp[,"Consensus"],temp[,"GIAB"])

tabbrep1 <- table(temp[,"GTtri1"],temp[,"GIAB"])

tabbrep2 <- table(temp[,"GTtri2"],temp[,"GIAB"])

tabbrep3 <- table(temp[,"GTtri3"],temp[,"GIAB"])

##############################################

## Performance indicators Consensus callset ##

# Accuracy:

(tabbcon[1,1]+ tabbcon[2,2]+ tabbcon[3,3])/ sum(tabbcon[1:3,])

# Recall:

(tabbcon[2,2]+ tabbcon[3,3]) / nrow(giab)

(tabbcon[2,2]+ tabbcon[3,3]) / 3238599

# Precision:

(tabbcon[2,2]+ tabbcon[3,3]) / sum(tabbcon[2:3,])

# F1-score = 2* Precision * Recall / (Precisioon + Recall)

## Performance indicators e.g. replicate 2 ##

Accuracy: (tabbrep2[1,1]+ tabbrep2[2,2]+ tabbrep2[3,3])/ sum(tabbrep2[1:3,])

Recall: (tabbrep2[2,2]+ tabbrep2[3,3]) / 3238599

Precision: (tabbrep2[2,2]+ tabbrep2[3,3]) / sum(tabbrep2[2:3,])

# F1-score = 2* Precision * Recall / (Precisioon + Recall)

################### MODEL 2 ##########################

############### LCA using poLCA package #######################

library(poLCA)

f <- cbind(GTtri1, GTtri2,GTtri3) ~ 1

m0 <- poLCA(formula=f, data = vcf, nclass = 3, nrep=50)

temp <- data.frame(temp, m0$predclass)

colnames(temp)<-c("GTtri1","GTtri2","GTtri3","GIAB", "Consensus", "LCA Classifier")

table(m0$predclass)

tabblca <- table(m0$predclass,datalca[,"GIAB"])

## Sort by increasing order of the latent class prevalence (= interpreted as 0/0, 1/1, 0/1)

o <- order(m0$P)

tabblca <- tabblca[o,]

## To match the same genotype category as in the colomns (GIAB)( =0/0, 0/1, 1/1)

tabblca <- tabblca[c(1,3,2),]

## Performance indicators LCA callset ##

# Accuracy:

(tabblca[1,1]+ tabblca[2,2]+ tabblca[3,3])/ sum(tabblca[1:3,])

# Recall:

(tabblca[2,2]+ tabblca[3,3]) / 3238599

# Precision:

(tabblca[2,2]+ tabblca[3,3]) / sum(tabblca[2:3,])

# F1-score = 2* Precision * Recall / (Precisioon + Recall)

################### MODEL 3 ###################

######## LCA Models with covariates ##########

###############################################

f3 <- cbind(GTtri1, GTtri2,GTtri3) ~ABmean

f4 <- cbind(GTtri1, GTtri2,GTtri3) ~ QD

f5 <- cbind(GTtri1, GTtri2,GTtri3) ~ MQ

f6 <- cbind(GTtri1, GTtri2,GTtri3) ~ QD + MQ

f7 <- cbind(GTtri1, GTtri2,GTtri3) ~ QD + ABmean

f8 <- cbind(GTtri1, GTtri2,GTtri3) ~ MQ + ABmean

m3 <- poLCA(formula=f3, data = vcf, nclass = 3, nrep=100)

m4 <- poLCA(formula=f4, data = vcf, nclass = 3, nrep=100)

m5 <- poLCA(formula=f5, data = vcf, nclass = 3, nrep=100)

m6 <- poLCA(formula=f6, data = vcf, nclass = 3, nrep=100)

m7 <- poLCA(formula=f7, data = vcf, nclass = 3, nrep=100)

m8 <- poLCA(formula=f8, data = vcf, nclass = 3, nrep=100)

############### Supplementary Table #########################

############ Evaluation of parameter estimation stability ########

recordlca <- function(f, n,data) {

mat <- matrix(NA,nrow = n, ncol = 4)

for (i in 1:n) {

gss.lc <- poLCA(formula = f, data = data, nclass = 3, maxiter = 3000)

mat[i,1] <- gss.lc$llik

o <- order(gss.lc$P,decreasing=T)

mat[i,-1] <- gss.lc$P[o]

print(i)

}

return(mat)

}

m3mat <- recordlca(f = f3, n = 1000, data = vcf)

m4mat <- recordlca(f = f4, n = 1000, data = vcf)

table(round(m3mat[,1], digits = 0))

table(round(m4mat[,1], digits = 0))

#####################################################################

### Summary Table of models #########

tabb <- data.frame(

c(m00[c("bic","time")],m0$P[order(m0$P,decreasing=T)]),c(m3[c("bic","time")],m3$P[order(m3$P,decreasing=T)]),

c(m4[c("bic","time")],m4$P[order(m4$P,decreasing=T)]),c(m5[c("bic","time")],m5$P[order(m5$P,decreasing=T)]),

c(m6[c("bic","time")],m6$P[order(m6$P,decreasing=T)]),c(m7[c("bic","time")],m7$P[order(m7$P,decreasing=T)]),

c(m8[c("bic","time")],m8$P[order(m8$P,decreasing=T)]))

### choose m4 with QD ##

LCA_QD <- m4$predclass

temp <-cbind(temp, LCA_QD)

colnames(temp)<-c("GTtri1","GTtri2","GTtri3","GIAB", "Consensus", "LCA Classifier", "LCA_QD")

tabblcaqd <- table(m4$predclass,temp[,"GIAB"])

## Sort by increasing order of the latent class prevalence (= interpreted as 0/0, 1/1, 0/1)

o <- order(m4$P)

tabblcaqd <- tabblcaqd[o,]

## To match the same genotype category as in the colomns (GIAB)( =0/0, 0/1, 1/1)

tabblcaqd <- tabblcaqd [c(1,3,2),]

## Performance indicators LCA callset ##

# Accuracy:

(tabblcaqd[1,1]+ tabblcaqd[2,2]+ tabblcaqd[3,3])/ sum(tabblcaqd[1:3,])

# Recall:

(tabblcaqd[2,2]+ tabblcaqd[3,3]) / 3238599

# Precision:

(tabblcaqd[2,2]+ tabblcaqd[3,3]) / sum(tabblcaqd[2:3,])

# F1-score = 2* Precision * Recall / (Precisioon + Recall)

#############################################################

############## Gaussian Mixture Model ######################

#############################################################

### "mclust" package

library(mclust)

m1 <- Mclust(data = vcf[,c("DPmean","ABmean","QD")],G =3)

temp <- cbind(temp, m1$classification)

colnames(temp)<-c("GTtri1","GTtri2","GTtri3","GIAB", "Consensus", "LCA Classifier", "LCA_QD","GMM")

tabgmm <- table(m1$classification, temp[,"GIAB"], useNA = "ifany")

## Sort by increasing order of the latent class prevalence (= interpreted as 0/0, 1/1, 0/1)

o <- order(m1$parameters$pro)

tabgmm <- tabgmm[o,]

## To match the same genotype category as in the colomns (GIAB)( =0/0, 0/1, 1/1)

tabgmm <- tabgmm[c(1,3,2),]

## Performance indicators of gaussian mixture model callset ##

# Accuracy:

(tabgmm[1,1]+ tabgmm[2,2]+ tabgmm[3,3])/ sum(tabgmm[1:3,])

# Recall:

(tabgmm[2,2]+ tabgmm[3,3]) / 3238599

# Precision:

(tabgmm[2,2]+ tabgmm[3,3]) / sum(tabgmm[2:3,])

# F1-score = 2* Precision * Recall / (Precisioon + Recall)

################# MODEL 4 ###########################

################## KAMILA ############################

######################################################

library(kamila)

datkamila <- vcf

datkamila[ which(datkamila[,"DPmean"] > 150), "DPmean"] <- 150

## Define covariables

conVar = data.frame(scale(datkamila[,c("DPmean","ABmean","QD")]))

catDf <- data.frame(as.factor(datkamila[,"GTtri1"]),as.factor(datkamila[,"GTtri2"]),as.factor(datkamila[,"GTtri3"]),as.factor(datkamila[,"blacklist"]))

mkamila <- kamila(conVar <- conVar, catFactor <- catDf, numClust = 3, numInit = 50)

temp <- cbind(temp, mkamila$finalMemb)

colnames(temp)<-c("GTtri1","GTtri2","GTtri3","GIAB", "Consensus", "LCA Classifier", "LCA_QD","GMM","Kamila")

tabbkam <- table(mkamila$finalMemb,temp[,"GIAB"],useNA = "ifany")

## Sort by increasing order of the latent class prevalence (= interpreted as 0/0, 1/1, 0/1)

o <- order(matrix(table(mkamila$finalMemb))[,1])

tabbkam <- tabbkam[o,]

## To match the same genotype category as in the colomns (GIAB)( =0/0, 0/1, 1/1)

tabbkam <- tabbkam[c(1,3,2),]

## Performance indicators of gaussian mixture model callset ##

#Accuracy:

(tabbkam[1,1]+ tabbkam[2,2]+ tabbkam[3,3])/ sum(tabbkam[1:3,])

#Recall:

(tabbkam[2,2]+ tabbkam[3,3]) / 3238599

#Precision:

(tabbkam[2,2]+ tabbkam[3,3]) / sum(tabbkam[2:3,])

# F1-score = 2* Precision * Recall / (Precisioon + Recall)

################# MODEL 5 ##############

############# RandomForest #############

#########################################

library(randomForest)

datrf <- vcf[,c("GTtri1","GTtri2","GTtri3","DPmean","ABmean","QD","blacklist")]

set.seed(70)

datrf<-datrf[sample(nrow(datrf),10000,replace = FALSE), ]

# model

rf.fit <- randomForest(x = datrf[,c(1:7)], y = NULL, ntree = 1000, proximity = TRUE, oob.prox = TRUE)

hclust.rf <- hclust(as.dist(1-rf.fit$proximity), method = "ward.D2")

rf.cluster = cutree(hclust.rf, k=3)

table(rf.cluster)

temp <- cbind(temp,rf.cluster)

colnames(temp)<-c("GTtri1","GTtri2","GTtri3","GIAB", "Consensus", "LCA Classifier", "LCA_QD",

"GMM","Kamila","RandomForest")

tabrf <- table(rf.cluster,temp[,"GIAB"],useNA = "ifany")

## Sort by increasing order of the latent class prevalence (= interpreted as 0/0, 1/1, 0/1)

o <- order(matrix(table(rf.cluster))[,1])

tabrf <- tabrf[o,]

## To match the same genotype category as in the colomns (GIAB)( =0/0, 0/1, 1/1)

tabrf <- tabrf[c(1,3,2),]

## Performance indicators of gaussian mixture model callset ##

#Accuracy:

(tabrf[1,1]+ tabrf[2,2]+ tabrf[3,3])/ sum(tabrf[1:3,])

#Recall:

(tabrf[2,2]+ tabrf[3,3]) / (10000 * nrow(giab)/nrow(vcf))

# (tabrf[2,2]+ tabrf[3,3]) / (10000 * 3238599/3351415)

#Precision:

(tabrf[2,2]+ tabrf[3,3]) / sum(tabrf[2:3,])

# F1-score = 2* Precision * Recall / (Precisioon + Recall)
